# Translation and cultural adaptation of the COVID-19 Yorkshire Rehabilitation Scale into German

**DOI:** 10.3389/fmed.2024.1401491

**Published:** 2024-08-30

**Authors:** Lisa Sperl, Tanja Stamm, Erika Mosor, Valentin Ritschl, Manoj Sivan, Kathryn Hoffmann, Brigitte Gantschnig

**Affiliations:** ^1^Institute of Outcomes Research, Medical University of Vienna, Vienna, Austria; ^2^Ludwig Boltzmann Institute for Arthritis and Rehabilitation, Vienna, Austria; ^3^Academic Department of Rehabilitation Medicine, University of Leeds, Leeds, United Kingdom; ^4^Department for Primary Care Medicine, Center for Public Health, Medical University of Vienna, Vienna, Austria; ^5^Institute of Occupational Therapy, School of Health Sciences, ZHAW Zurich University of Applied Sciences, Winterthur, Switzerland; ^6^Department of Rheumatology and Immunology, University Hospital, Inselspital, University of Bern, Bern, Switzerland

**Keywords:** long-COVID, long-term consequences, post-acute sequelae of SARS-CoV-2 (PASC), rehabilitation, patient-reported outcomes

## Abstract

**Background:**

Experts estimate that in up to 10% of the infected, SARS-CoV-2 would cause persistent symptoms, activity limitations and reduced quality of life. Referred to as long COVID, these conditions might, in the future, specifically impact German-speaking countries due to their higher rates of unvaccinated people compared to other Western countries. Accurate measurement of symptom burden and its consequences is needed to manage conditions such as long COVID, and several tools have been developed to do so. However, no patient-reported instrument existed in the German language at the time of writing.

**Objective:**

This study, therefore, aimed to develop a German version of the COVID-19 Yorkshire Rehabilitation Scale (C19-YRS).

**Methods:**

We conducted a translation and qualitative evaluation, including cultural adaptation, of the C19-YRS and assessed its face validity. After creating a preliminary version, 26 individuals (14 women [53%]) participated in cognitive interviews (January 2022 to March 2022). Using cognitive debriefing interviews, we ensured the content’s comprehensibility. The matrix-framework method guided the qualitative data analysis.

**Results:**

Compared to the original English version, adaptations were necessary, resulting in changes to the introductory text, while the items for recording persistent symptoms were hardly changed.

**Conclusion:**

The German version of the C19-YRS is expected to support standardized long COVID care.

## 1 Introduction

Experts estimate that in up to 10% of the infected, the severe acute respiratory syndrome coronavirus type 2 (SARS-CoV-2) causes persistent symptoms, activity limitations and reduced quality of life (1, 2). Literature refers to these conditions as long COVID and defines them as a combination of manifestations such as fatigue, exhaustion, decreased physical endurance, post-exertional malaise, breathing difficulties, anxiety, depression, posttraumatic stress disorder or pain, lasting for more than 4 weeks after the infection (3–9). Long COVID can affect people of all ages, regardless of whether they had a severe or mild course of disease (5, 9, 10). Vaccination tends to lead to a risk reduction regarding long COVID symptoms (1, 11–13), whereas reinfections seem to cumulatively increase the risk (14).

Long COVID could particularly impact German-speaking countries in Europe, where the number of unvaccinated people is high (15) as well as reinfection rate due to the lack of primary prevention since spring 2023. Accurate measurement of symptom burden and its impact on people’s daily lives is needed to manage this condition effectively, and rapid assessment is necessary to fully assess patients’ problems and enable targeted multidisciplinary intervention (16). To date, the C19-YRS is the only tool that provides exemplary ideas for patient-centered management and interventions based on the severity of problems reported in the screening tool (17). Completing the C19-YRS over time provides a comprehensive overview of a patient’s progress, whether their condition is improving, worsening or fluctuating, which then also supports goal setting and the planning of patient-centered therapeutic interventions. Translating and adapting a multi-professional, COVID-19-specific assessment tool such as the C19-YRS can facilitate comprehensive assessment and intervention counseling, potentially improving standardized care for people with persistent symptoms after acute COVID-19 infection (17). No patient-reported instrument existed in the German language at the time of writing. Therefore, this study aimed to develop a German version of the COVID-19 Yorkshire Rehabilitation Scale (C19-YRS).

## 2 Materials and methods

We conducted a translation and qualitative evaluation, including cultural adaptation, of the C19-YRS into German and assessed its face validity.

### 2.1 COVID-19 Yorkshire Rehabilitation Scale (C19-YRS)

The COVID-19 Yorkshire Rehabilitation Scale is a 22-item patient-reported outcome measure for assessing and monitoring long COVID symptoms and was the first long COVID-specific scale reported in the literature (18). Considering psychometric properties, the English version of the C19-YRS showed good internal consistency, and scaling and targeting assumptions were satisfied (19).

Information collected includes 16 symptoms (including shortness of breath, persistent cough, fatigue, pain or discomfort, cognitive problems, anxiety, depression, symptoms of posttraumatic stress disorder [PTSD], palpitations, dizziness, weakness, and sleep problems) as well as their impact on five areas of activities and participation (including communication, mobility, personal care, activities of daily living, and social life) (see Supplementary Appendix 1). The patient is asked to rate each symptom or functional ability on a scale from 0 to 10 (0 being not present and ten being most severe and life-disturbing) (18). The C19-YRS is recommended for initial assessment, at 6 weeks, and at 6 months for follow-up and includes a self-report version. In a self-reported screening tool, outcomes are reported directly without interference from clinicians or health professionals (20), which supports people’s active participation in the decision-making process regarding their health care (21). For this reason, it was decided to first translate the self-reported version of the C19-YRS within the scope of this paper.

### 2.2 Translation and cross-cultural adaptation

First, the authors contacted the team of developers of the original C19-YRS, who granted permission to translate the self-report version of the C19-YRS into German (Austria). The translation process followed five steps according to the guideline established by Beaton et al. (22) namely (1) initial translation, (2) synthesis of translations, (3) back translation, (4) expert committee, and (5) review of preliminary version. In the first step (1), two translators each produced a German version of the C19-YRS (T1&T2). In a second step (2), a third person, who also has expertise in research and translation processes, helped synthesis these translations (T1&T2) into a new version (T12). At this point, the translation team added a step to Beaton’s guideline, as a person who is not a native German speaker reviewed the synthesized version to ensure that it was easy to understand. Then, in the third step (3), two English first language translators who were not familiar with the screening tool worked from the T12 translation and produced two back translations (BT1&BT2). In the fourth step (4), an expert committee (methodologist [TS], linguist [LD], translators) reviewed all reports to reach a consensus and jointly produced a preliminary version. Beaton et al. (22) suggest that individuals from the target group should subsequently complete the preliminary version to test understanding of the items. For this purpose, the authors then decided to apply cognitive interviewing methods in the last step of the translation and adaptation process (5) (23). In addition, the cross-cultural adaptation of a health status screening instrument usually involves assessing validity and reliability (24). In the context of this study, an initial aspect of content, validity face validity, which assesses the extent to which a measurement instrument adequately reflects the construct being measured (25), was chosen.

### 2.3 Participants and sampling

For the cognitive interviews, the first author purposively recruited patients from Austrian rehabilitation centers participating in the Austrian long COVID registry. Participation was open to people who had been diagnosed with COVID-19 infection, who were experiencing long-term symptoms following a COVID-19 infection, who were at least 18 years old, who understood and spoke sufficient German and who agreed to participate. This study involving human participants was reviewed and approved by the ethics committee of the Medical University Vienna as part of the Austrian Long COVID registry Project (EK 1591/2021). Written informed consent to participate in this study was provided by participants.

The Austrian long COVID registry is a nationwide registry, supported by Gesundheit Österreich GmbH, the Ministry of Health, Medical University of Vienna, Austria Health Insurance Fund, Danube University Krems and two Ludwig Boltzmann Institutes. The overall aim of the registry is to assess the disease course of post/long-COVID-19, evaluate its impact on quality of life on functional capacity, and, e.g., assess the interventions offered (26). Individuals who are referred to one of the participating centers - from primary care centers to rehabilitation centers - and who meet the above inclusion criteria will be asked to complete an online questionnaire covering the above objectives in self-report form and will then be registered in this way.

### 2.4 Data collection

The authors chose the two most common cognitive interviewing techniques for data collection, thinking aloud and probing (27). The interviews were conducted by the first author and took place either in person at the rehabilitation center or by telephone. Participants were instructed to express what came to their mind (“thinking aloud”) while concurrently completing the C19-YRS (23), and in addition they answered probes retrospectively (28) (see Supplementary Appendix 3).

Typically, 10 to 50 interviews are conducted and analyzed in cognitive interviewing studies (29). Since no further new aspects emerged after 26 interviews, the author ended the data collection at this point as she assumed that thematic saturation was reached. Thematic saturation is achieved when further observations and analyzes do not yield any newer topics (30).

### 2.5 Data analysis

The authors used a method of analysis called framework, a matrix-based approach to manage qualitative data (31, 32). They entered summaries of individual interviews into a series of grids. Domains of inquiry, in this case, each question and text of the C19-YRS were in the same order as in the test questionnaire. This approach allowed the authors to review the data collected systematically (33).

#### 2.5.1 Descriptive analysis

Next, the authors conducted a descriptive analysis to understand how participants interpreted the test questions and identified factors that influenced interpretation and responses. Familiarity with the data was ensured by reading the matrices and taking notes at each step to record ideas that emerged and were considered helpful for moving forward. The range of responses for each question was noted and then categorized in the next step to identify similarities and differences as participants may have interpreted to test questions differently (34).

#### 2.5.2 Explanatory analysis

Then, the authors conducted an explanatory analysis to understand how these potential problems arose. First, they identified patterns in the data, and then these patterns were linked to, for example, participant understanding or response, which helped identify mechanisms by which problems emerged. The next step was to develop explanations for the patterns, which relied on participants’ accounts captured in the cognitive interviews, whether through individual utterances or observations (34). The authors assessed whether the problem could potentially affect the quality of the data extracted from the Screening tool and made the necessary changes to the preliminary version of the C19-YRS (34).

During translation and cultural adaptation, translators wrote a report for each step they were involved. The expert committee then produced a preliminary version for the first author to use for pre-testing in the target population. All translators tried to stay close to the original version in this translation and adaptation process, but some changes were also necessary due to cultural differences. After completing the descriptive and explanatory analysis, the preliminary version was revised and sent to two health professionals experienced in working with people suffering from persistent symptoms after acute COVID-19 infection. Similar to peer-reviewing, their comments were then incorporated when creating the final Austrian-German version of the C19-YRS.

## 3 Results

First, the translators decided to expand the subtitle to “Self Report Version” to make the objective of the screening tool sufficiently clear from the outset, although the original English title of the C19-YRS, “COVID-19 Yorkshire Rehabilitation Scale (C19-YRS)”, was retained. Therefore, they decided to expand the subtitle to “Self-Assessment Questionnaire for the Assessment of Persistent COVID-19 Symptoms”. Second, the original introductory text stated, “Your answers will be recorded in your clinical record”. The translators recognized they could not generally claim that the data collected would be recorded in clinical records, so this was deleted. Third it states, “If you can’t remember, just indicate “don’t know” Since there was no box for “don’t know” where this could have been written, this was erased because the translators felt it could be misleading and confusing.

The first author then used this preliminary version to conduct the cognitive interviews. Twenty-six participants agreed to take part in the cognitive interviews. All of these participants were still suffering from persistent symptoms at the time of recruitment and thus constituted the target population of the C19-YRS. Characteristics of the study population are presented in Table 1.

**TABLE 1:** Characteristics of participants.

Characteristics		*n* = 26 (100%)
Sex, *n*	Female, *n* (%)	14 (53.8%)
	Male, *n* (%)	12 (46.2%)
Native in German	Yes, *n* (%)	21 (80.8%)
	No, *n* (%)	5 (19.2%)
Age, years	Mean ( ± SD)	51.5 (±10.7)
	Median [25–75%]	51.0 [43.8 – 60.0]
1. COVID-19 infection	October 2020, *n* (%)	3 (11.5%)
	November 2020, *n* (%)	3 (11.5%)
	December 2020, *n* (%)	1 (3.8%)
	January 2021, *n* (%)	1 (3.8%)
	February 2021, *n* (%)	1 (3.8%)
	March 2021, *n* (%)	4 (15.4%)
	April 2021, *n* (%)	3 (11.5%)
	May 2021, *n* (%)	1 (3.8%)
	September 2021, *n* (%)	2 (7.7%)
	October 2021, *n* (%)	2 (7.7%)
	November 2021, *n* (%)	5 (19.2%)
2. COVID-19 infection	January 2022, *n* (%)	1 (3.8%)
	February 2022, *n* (%)	1 (3.8%)
	No second Infection, *n* (%)	24 (92.3%)
Months since 1. COVID-19 infection	Mean ( ± SD)	10.54 (±4.81)

Cognitive interviews lasted from 20 to 30 min, whether the interviewer conducted the interviews via telephone (*n* = 10) or on site (*n* = 16), while participants were filling out the pre-final version of the C19-YRS. The results of the interviews were primarily based on statements made by participants during the think aloud process, supported by the probes administered, as well as observations made from the interviewer. Observations were limited in telephone interviews, however, think aloud was more sufficient on the telephone as the participants appeared to talk the interviewer through each question more than those on site. The sample size of 26 seems appropriate, as no new topics emerged.

Questions 1-3

Q1 to 3 asked for the patient code, date and time, first under the heading. Fourteen of twenty-six participants skipped completing Q1 to 3 without further comment. The author observed that respondents were more likely to start with reading the introduction and therefore overlook these short questions. She suggested that these questions be listed after the introduction, and before the opening questions, and changed the location of Q1 to 3 accordingly.

Question 5 (Q5)

When completing Q5, which asked for health care services used for treatment of COVID-19 symptoms, eight participants wondered whether this question also meant specific health services such as rehabilitation centers and pulmonary physicians. They stated that the term “other health services” was not specific enough, therefore, the author decided to add other examples besides general practitioner (GP) that were mentioned by participants when thinking aloud (e.g., pulmonary physicians, 1450 Corona hotline in Austria). In addition, two participants asked whether Q5 meant the time of the acute infection or the time after, which could also have an influence on the answers, as the responses may vary depending on the requested time. It was clear from the outset of the original questionnaire that this question asked about treatment during the acute infection, so the author adjusted the translation accordingly.

Question 6 (Q6)

Eight out of twenty-six respondents were observed to complete Q6, which measures the extent of breathlessness, incorrectly because the answer to the question was misplaced. Three respondents additionally asked for help in completing it. The author observed that the visual representation of the question was misleading in this part of the questionnaire, as was the case with Q1 to 3, and therefore adapted this question to the visual appearance of the others (see Table 2).

**TABLE 2:** Changes in the visual representation of Question 6.

C19-YRS Domain	Preliminary version			English version	
1. Atemlosigkeit/ Kurzatmigkeit	Auf einer Skala von 0 bis 10, wie schwer würden Sie eine (eventuell vorhandene) Atemlosigkeit/ Kurzatmigkeit einschätzen? Bewerten Sie den Schweregrad dieses Problems (zwischen 0 - nicht vorhanden und 10 - schwerwiegend und Ihr Leben beeinträchtigend). (keine Antwort (k/a), wenn Sie die unten angeführten Tätigkeiten nicht ausüben)	Jetzt	Vor-Covid	On a scale of 0 to 10, how severe would you rate breathlessness/shortness of breath (if present)? Rate the severity of this problem (between 0 - nonexistent and 10 - severe and affecting your life). (no answer (N/A) if you do not perform the activities listed below).	
	In Ruhe	0–10:____	0–10:____	At rest	
	Beim Anziehen	0–10:____ k/a ☐	0–10:____ k/a ☐	On dressing yourself?	
	Beim Treppen hinaufsteigen	0–10:____ k/a ☐	0–10:____ k/a ☐	On walking up a flight of stairs?	
**C19-YRS Domain**	**Final version**			**English version**	
1. Atemlosigkeit/ Kurzatmigkeit	Auf einer Skala von 0 bis 10, wie schwer würden Sie eine (eventuell vorhandene) Atemlosigkeit/ Kurzatmigkeit einschätzen? Bewerten Sie den Schweregrad dieses Problems (zwischen 0 - nicht vorhanden und 10 - schwerwiegend und Ihr Leben beeinträchtigend). (keine Antwort (k/a), wenn Sie die unten angeführten Tätigkeiten nicht ausüben)			On a scale of 0 to 10, how severe would you rate breathlessness/shortness of breath (if present)? Rate the severity of this problem (between 0 - nonexistent and 10 - severe and affecting your life). (no answer (N/A) if you do not perform the activities listed below).	
		Vor-COVID	Jetzt	Pre-COVID	Now
	In Ruhe	0–10:____	0–10:____	At rest	
	Beim Anziehen	0–10:____ k/a ☐	0–10:____ k/a ☐	While dressing	
	Beim Treppen hinaufsteigen	0–10:____ k/a ☐	0–10:____ k/a ☐	When going up stairs	

Question 15 (Q15)

All participants agreed that Q15, which was screening post-traumatic stress disorder (PTSD), had to be read repeatedly to be understood while some also asked the interviewer for a verbal explanation. Four other participants interpreted the question as referring only to people who were in the hospital. The author concluded that the wording of the question was flawed and changed the question according to the verbal explanations she gave to the participants during the interviews, which were well understood.

Question 20 (Q20)

Nine participants indicated that Q20, evaluating the social role, was not easy to understand and required a verbal explanation from the author. Participants suggested that the question be reworded and Q20, like Q15 above, was rephrased according to the author’s verbal explanation.

Question 21 (Q21)

Eight participants mentioned that Q21, requesting information on employment, was not clear, and they said that the answer choices provided did not correspond to the question asked. Therefore, the author tried to rephrase the original question to connect to the answers more appropriately.

According to every participant, the screening instrument has generally worked well for capturing their long COVID condition. Participants indicated that the C19-YRS was clear, feasible, user-friendly, and appropriate. Therefore, the authors would like to state that it appears that the face validity in the C19-YRS is good.

Following the results of the cognitive interview data analysis, the authors made changes to the preliminary version of the C19-YRS. The changes to the preliminary version were then proofread by two health professionals who work with patients with persistent symptoms after acute COVID-19 infection. After the authors incorporated their comments, which were mainly wording recommendations, a final version was prepared (see Supplementary Appendix 2). Table 3 illustrates the changes made from the preliminary to the final version, including feedback from both participants and health professionals.

**TABLE 3:** Changes from the preliminary version to the final version of the C19-YRS.

C19-YRS Domain	Preliminary version	Final version	English version
Q1, Q2, Q3	Patient*innen Code: Ausfülldatum (tt.mm.jjjj): Uhrzeit (hh:mm): (before introduction text)	Patient*innen ID: Ausfülldatum (tt.mm.jjjj): Uhrzeit (hh:mm): (after introduction text)	Patient ID: Date filled in (dd.mm.yyyy): Time (hh:mm): (after introduction text)
Q5	Haben Sie andere Gesundheitsdienstleistungen zur Behandlung von COVID-19 Symptomen in Anspruch genommen (z.B. Allgemeinmediziner*in/ Hausärzt*in)? Ja ☐ Nein ☐ Details:	Haben Sie andere Gesundheitsdienstleistungen zur Behandlung von COVID-19 Symptomen in Anspruch genommen (z.B. Hausärzt*in, Lungenfachärzt*in, 1450 Corona Hotline)? Ja ☐ Nein ☐ Könnten Sie diese bitte konkretisieren:	Have you used other health care services to treat COVID-19 symptoms (e.g., primary care physician*, pulmonary specialist*, 1450 Corona Hotline)? Yes ☐ No ☐ Could you please be more specific:
Q6	See Table 2	See Table 2	See Table 2
Q15	a) Hatten Sie irgendwelche ungewollten Erinnerungen an Ihre Krankheit oder Ihren Krankenhausaufenthalt, während Sie wach waren, also nicht im Schlaf? Ja ☐ Nein ☐ b) Hatten Sie unangenehme Träume über Ihre Krankheit oder Ihren Krankenhausaufenthalt? Ja ☐ Nein? c) Haben Sie versucht, Gedanken oder Gefühle über Ihre Krankheit oder die Aufnahme ins Krankenhaus zu vermeiden? Ja ☐ Nein ☐	a) Hatten Sie irgendwelche unangenehmen Erinnerungen an Ihre Krankheit oder Ihren Krankenhausaufenthalt, während Sie wach waren, also nicht im Schlaf? Ja ☐ Nein ☐ b) Hatten Sie unangenehme Träume über Ihre Krankheit oder Ihren Krankenhausaufenthalt? Ja ☐ Nein? c) Haben Sie versucht, Gedanken oder Gefühle über Ihre Krankheit oder die Aufnahme ins Krankenhaus zu vermeiden? Ja ☐ Nein ☐	Did you have any unwanted memories of your illness or hospitalization while you were awake, that is, not asleep? Yes ☐ No ☐ b) Did you have any unpleasant dreams about your illness or hospitalization? Yes ☐ No? c) Have you tried to avoid thoughts or feelings about your illness or hospital admission? Yes ☐ No ☐
Q20	Wie schwerwiegend sind auf einer Skala von 0 bis 10 die Probleme, die Sie bei der Betreuung von Familienmitgliedern und/oder im Kontakt mit Freund*innen haben, die mit Ihrer Krankheit zusammenhängen (und nicht auf die COVID-19 Maßnahmen zur sozialen Distanzierung/ Lockdown zurückzuführen sind)? 0 bedeutet keine Probleme, 10 bedeutet schwerwiegende Probleme	Wie schwerwiegend sind auf einer Skala von 0 bis 10 die Probleme, die Sie zum Beispiel im Kontakt mit Familienmitgliedern oder mit Freund*innen haben? Gibt es hier Einschränkungen in ihrem sozialen Leben, die mit Ihren anhaltenden Symptomen zusammenhängen (und nicht auf die COVID-19 Maßnahmen zur sozialen Distanzierung/ Lockdown zurückzuführen sind)? 0 bedeutet keine Probleme, 10 bedeutet schwerwiegende Probleme	On a scale of 0 to 10, how severe are the problems you have, for example, in contact with family members or friends? Are there any limitations in your social life that are related to your persistent symptoms (and not due to the COVID-19 social distancing/lockdown measures)? 0 means no problems, 10 means severe problems.
Q21	In welchem Beschäftigungsverhältnis stehen Sie? Hat Ihre Krankheit Ihre Fähigkeit beeinträchtigt, Ihrer üblichen Arbeit nachzugehen? Beruf: Beschäftigungsstatus vor der Covid-19 Pandemie: Beschäftigungsstatus vor Ihrer Covid-19 Erkrankung: Aktueller Beschäftigungsstatus:	Wie ist ihr aktueller Beschäftigungsstatus? Hat Ihre Krankheit Ihre Fähigkeit beeinträchtigt, Ihrer üblichen Arbeit nachzugehen? Beruf: Beschäftigungsstatus vor der Covid-19 Pandemie: Beschäftigungsstatus vor Ihrer Covid-19 Erkrankung: Aktueller Beschäftigungsstatus:	What is your current employment status? Has your illness affected your ability to perform your usual work? Occupation: Employment status before the Covid-19 pandemic: Employment status prior to your Covid-19 illness: Current employment status:

## 4 Discussion

The present work is the first German version of the C19-YRS, a screening tool for long COVID. To date, this appears to be the only translation of a COVID-19-specific assessment or screening tool into German. Based on a rigorous translation and cross-cultural adaptation process that included 26 cognitive interviews with patients and feedback from health professionals working in the field of long COVID, as well as the evaluation of its face validity, a German version was created. Overall, this study emphasizes that the translated version of the C19-YRS appears suitable to assess persistent symptoms and to support establishment of standardized care in German speaking countries.

The final German C19-YRS has been carefully reviewed and compared with the original, as equivalence between the original and the translated version is considered important (35). No relevant differences were found that could affect the use of the C19-YRS in any way. Adjustments were necessary to adequately reflect the context in which the screening instrument is embedded. These inconsistencies primarily led to changes in the introductory text of the C19-YRS and the items recording the persistent symptoms were hardly affected.

However, instrument translation is usually accompanied by a change in context (25, 36). But compared to previous studies in which researchers reported that translation and cultural adaptation of assessments often lead to alteration in meaning and even deletion of items (37, 38), changes in the case of this study are marginal. Although the current work could be seen as an unproblematic adaptation process with only few changes, other explanations should also be discussed. As such, perhaps the premature state of current research on long COVID and the resulting limited knowledge of individuals affected and health professionals involved in their care (39) has actually restricted more critical examination of the C19-YRS. Widely varying descriptions and definitions of long COVID continue to appear in the literature, as well as in everyday language. With primary studies and reviews appearing at a rapid pace, current findings are inevitably associated with methodological differences and limitations. To improve our knowledge of long COVID, well-designed prospective studies are needed to establish long COVID definitions, accurate differentiation of symptoms, and appropriate treatment of this emerging condition (39). The increase in knowledge could then lead to a more critical review and adaptation process with the C19-YRS at a later date, possibly involving even greater significant changes.

The evaluation of the face validity was also rather superficial compared to other cross-sectional studies focusing on translation and cross-cultural adaptation (40, 41). Further investigation of psychometric properties, including internal consistency, already demonstrated in the original version (19), should be considered in future studies of the Austrian-German version of the C19-YRS.

## 5 Methodological considerations

The combination of different data sources and methods, as well as the involvement of numerous researchers in the translation process, in the sense of triangulation, enriched the data and reduced bias (42, 43). According to Collins (23), a sufficient number of participants were employed in this study, aiming to verify that the participants’ understanding of the questionnaire items was in line with the intended meaning. The sample was balanced, such that the participants represented both genders, a variety of age groups, and both native speakers and non-native speakers. Peer review by health professionals was also beneficial, as their expertise was particularly helpful in adapting this comprehensive assessment.

Nevertheless, this is a report of a small-scale study. In comparison, Beaton et al. (22) suggests 30–40 participants for the pre-test. This study should be replicated in a bigger sample, including a population characterized by different treatment experiences. In this case, only individuals who were already assigned to a rehabilitation facility, that can currently be considered an essential part of treatment for long COVID in Austria, participated in this study. Also, the fact that the interviews were not recorded and transcribed could lead to a lower credibility of the results. The interviews were conducted by the first author and predominantly evaluated by her. As a control, interviews could have been conducted and evaluated by other researches as well. A high agreement would have been a sign for trustworthiness (44), as in the study of Friedli and Gantschnig (37). Content validity and internal consistency should be considered for further investigations (26), as face validity, often giving a first impression without going into too much detail, is overall a very subjective assessment (45).

## 6 Conclusion

In conclusion, this study resulted in a German version of the C19-YRS. It is expected that the provision of this multi-professional screening tool will support the initial assessment of persistent symptoms, and the establishment of standardized care pathways in Austria. First, however, the psychometric properties should be further explored. Then, the efforts of the broader multi-professional rehabilitation team will be essential to ensure that this screening tool is successfully used in practice.

## Data Availability

The raw data supporting the conclusions of this article will be made available by the authors, without undue reservation.
