# Supplementary material for: Translation and cultural adaptation of the COVID-19 Yorkshire Rehabilitation Scale into German
Source: Front Med (Lausanne). 2024 Aug 30;11:1401491. doi: 10.3389/fmed.2024.1401491 (PMC11409085; doi:10.3389/fmed.2024.1401491)
Supplement: Supplementary file 1 [file Data_Sheet_1.pdf]

C19-YRS original version

COVID-19 Yorkshire Rehabilitation Scale (C19-YRS)

Self-report version

Patient name:

NHS number:

Date: Time:

We are getting in touch with people who have persistent health problems after having had a diagnosis of COVID-19 (coronavirus disease). The purpose of this questionnaire is to find out if you are experiencing problems related to your recent illness with COVID-19. Your responses will be recorded in your clinical notes. We will use this information to monitor your symptoms, offer treatments and assess response to treatment.

This questionnaire will take around 15 minutes. If there are any topics you don't want to talk about you can chose not to respond.

Do you consent for this information to be used for audit and research as well ? **Yes** ☐ **No** ☐

Opening questions:

Have you had any medical problems related to COVID-19 that needed hospital admission? **Yes** ☐ **No** ☐<br>Details:
Have you used any other health services to manage COVID-19 symptoms (e.g., your GP?) **Yes** ☐ **No** ☐<br>Details:

Please respond to the below questions to the best of your knowledge.

'Now' refers to how you feel now/this week.

"Pre-COVID" refers to how you were feeling prior to contracting the illness.

If you are unable to recall this, just state 'dont know'

1. Breathlessness	On a scale of 0-10, with 0 being not breathless at all, and 10 being extremely breathless, how breathless are you:<br>(n/a if you do not perform this activity)	Now	Pre-Covid
	a) At rest?	0-10: ____	0-10: ____
	b) On dressing yourself?	0-10: ____<br>n/a ☐	0-10: ____<br>n/a ☐
	c) On walking up a flight of stairs?	0-10: ____<br>n/a ☐	0-10: ____<br>n/a ☐

2. Cough/ throat sensitivity/ voice change	Have you got any of the below symptoms that is new since contracting the illness?<br>• cough/ throat sensitivity **Yes** ☐ **No** ☐<br>• voice change **Yes** ☐ **No** ☐<br>• noisy breathing **Yes** ☐ **No** ☐<br><br>Which of these three is the worst symptom -<br><br>Rate the severity of this problem (0 being not present, 10 being severe and life disturbing)<br>Now: **0** ☐ **1** ☐ **2** ☐ **3** ☐ **4** ☐ **5** ☐ **6** ☐ **7** ☐ **8** ☐ **9** ☐ **10** ☐<br>Pre-Covid: **0** ☐ **1** ☐ **2** ☐ **3** ☐ **4** ☐ **5** ☐ **6** ☐ **7** ☐ **8** ☐ **9** ☐ **10** ☐
3. Swallowing/ nutrition	Are you having difficulties eating, drinking or swallowing such as coughing, choking or avoiding any food or drinks? **Yes** ☐ **No** ☐<br>Rate the severity of swallowing problem (0 being no symptom, 10 being severe and life disturbing)<br>Now: **0** ☐ **1** ☐ **2** ☐ **3** ☐ **4** ☐ **5** ☐ **6** ☐ **7** ☐ **8** ☐ **9** ☐ **10** ☐<br>Pre-Covid: **0** ☐ **1** ☐ **2** ☐ **3** ☐ **4** ☐ **5** ☐ **6** ☐ **7** ☐ **8** ☐ **9** ☐ **10** ☐<br><br>Are you or your family concerned that you have ongoing weight loss or any ongoing nutritional concerns as a result of Covid-19? **Yes** ☐ **No** ☐
4. Fatigue	Do you become fatigued more easily compared to before your illness? **Yes** ☐ **No** ☐<br>Rate the severity of fatigue (0 being not present, 10 being severe and life disturbing)<br>Now: **0** ☐ **1** ☐ **2** ☐ **3** ☐ **4** ☐ **5** ☐ **6** ☐ **7** ☐ **8** ☐ **9** ☐ **10** ☐<br>Pre-Covid: **0** ☐ **1** ☐ **2** ☐ **3** ☐ **4** ☐ **5** ☐ **6** ☐ **7** ☐ **8** ☐ **9** ☐ **10** ☐
5. Continence	Since your illness are you having any <u>new</u> problems with:<br>• controlling your bowel **Yes** ☐ **No** ☐<br>• controlling your bladder **Yes** ☐ **No** ☐<br><br>Which of these two is the worst symptom -<br><br>Rate the severity of this problem (0 being not present, 10 being severe and life disturbing)<br>Now: **0** ☐ **1** ☐ **2** ☐ **3** ☐ **4** ☐ **5** ☐ **6** ☐ **7** ☐ **8** ☐ **9** ☐ **10** ☐<br>Pre-Covid: **0** ☐ **1** ☐ **2** ☐ **3** ☐ **4** ☐ **5** ☐ **6** ☐ **7** ☐ **8** ☐ **9** ☐ **10** ☐
6. Pain/ discomfort	Have you got any pain that is new since contracting the illness? **Yes** ☐ **No** ☐<br>If Yes,<br>• chest pain **Yes** ☐ **No** ☐<br>• joint pain **Yes** ☐ **No** ☐<br>• muscle pain **Yes** ☐ **No** ☐<br><br>• headache **Yes** ☐ **No** ☐<br>• abdominal pain **Yes** ☐ **No** ☐<br>• other pain **Yes** ☐ **No** ☐<br><br>Within the last week, which of the these was the worst problem –<br><br>Rate the severity of this problem (0 being no pain or discomfort, 10 being severe and life disturbing pain)<br>Now: **0** ☐ **1** ☐ **2** ☐ **3** ☐ **4** ☐ **5** ☐ **6** ☐ **7** ☐ **8** ☐ **9** ☐ **10** ☐<br>Pre-Covid: **0** ☐ **1** ☐ **2** ☐ **3** ☐ **4** ☐ **5** ☐ **6** ☐ **7** ☐ **8** ☐ **9** ☐ **10** ☐
7. Cognition	Since your illness have you had new or worsened difficulty with:<br>• concentrating? **Yes** ☐ **No** ☐<br>• short term memory? **Yes** ☐ **No** ☐<br>• planning? **Yes** ☐ **No** ☐

	Which of these three is the worst symptom –<br><br>Rate the severity of this problem (0 being not present, 10 being severe and life disturbing)<br>Now: **0** ☐ **1** ☐ **2** ☐ **3** ☐ **4** ☐ **5** ☐ **6** ☐ **7** ☐ **8** ☐ **9** ☐ **10** ☐<br>Pre-Covid: **0** ☐ **1** ☐ **2** ☐ **3** ☐ **4** ☐ **5** ☐ **6** ☐ **7** ☐ **8** ☐ **9** ☐ **10** ☐
8. Anxiety	On a 0-10 scale, how severe is any anxiety you are experiencing?<br>0 means I am not anxious, 10 means I am extremely anxious.<br>Now: **0** ☐ **1** ☐ **2** ☐ **3** ☐ **4** ☐ **5** ☐ **6** ☐ **7** ☐ **8** ☐ **9** ☐ **10** ☐<br>Pre-Covid: **0** ☐ **1** ☐ **2** ☐ **3** ☐ **4** ☐ **5** ☐ **6** ☐ **7** ☐ **8** ☐ **9** ☐ **10** ☐
9. Depression	On a 0-10 scale, how severe is any depression you are experiencing?<br>0 means I am not depressed, 10 means I have extreme depression.<br>Now: **0** ☐ **1** ☐ **2** ☐ **3** ☐ **4** ☐ **5** ☐ **6** ☐ **7** ☐ **8** ☐ **9** ☐ **10** ☐<br>Pre-Covid: **0** ☐ **1** ☐ **2** ☐ **3** ☐ **4** ☐ **5** ☐ **6** ☐ **7** ☐ **8** ☐ **9** ☐ **10** ☐<br>Are you currently having thoughts about harming yourself in any way? **Yes** ☐ **No** ☐
10. PTSD screen	a) Have you had any unwanted memories of your illness or hospital admission whilst you were awake, so not counting dreams? **Yes** ☐ **No** ☐<br><br>b) Have you had any unpleasant dreams about your illness or hospital admission? **Yes**☐ **No** ☐<br><br>c) Have you tried to avoid thoughts or feelings about your illness or hospital admission? **Yes** ☐ **No** ☐<br><br>Rate the severity of these stress problems (0 being not present, 10 being severe and life disturbing)<br>Now: **0** ☐ **1** ☐ **2** ☐ **3** ☐ **4** ☐ **5** ☐ **6** ☐ **7** ☐ **8** ☐ **9** ☐ **10** ☐<br>Pre-Covid: **0** ☐ **1** ☐ **2** ☐ **3** ☐ **4** ☐ **5** ☐ **6** ☐ **7** ☐ **8** ☐ **9** ☐ **10** ☐
11. Communication	Since your illness have you had new or worsened difficulty with communication/word finding difficulty/ understanding others ? **Yes** ☐ **No** ☐<br>Rate the severity of communication problem (0 being not present, 10 being severe and life disturbing)<br>Now: **0** ☐ **1** ☐ **2** ☐ **3** ☐ **4** ☐ **5** ☐ **6** ☐ **7** ☐ **8** ☐ **9** ☐ **10** ☐<br>Pre-Covid: **0** ☐ **1** ☐ **2** ☐ **3** ☐ **4** ☐ **5** ☐ **6** ☐ **7** ☐ **8** ☐ **9** ☐ **10** ☐
12. Mobility	On a 0-10 scale, how severe are any problems you have in walking about?<br>*Or moving about if you normally walk using aids*<br>0 means no problems, 10 means severe or completely unable to walk about.<br>Now: **0** ☐ **1** ☐ **2** ☐ **3** ☐ **4** ☐ **5** ☐ **6** ☐ **7** ☐ **8** ☐ **9** ☐ **10** ☐<br>Pre-Covid: **0** ☐ **1** ☐ **2** ☐ **3** ☐ **4** ☐ **5** ☐ **6** ☐ **7** ☐ **8** ☐ **9** ☐ **10** ☐
13. Personal-Care	On a 0-10 scale, how severe are any problems you have in personal cares such as using the toilet, washing and dressing yourself?<br>0 means no problems, 10 means completely unable to do or fully dependent on others to help.<br>Now: **0** ☐ **1** ☐ **2** ☐ **3** ☐ **4** ☐ **5** ☐ **6** ☐ **7** ☐ **8** ☐ **9** ☐ **10** ☐<br>Pre-Covid: **0** ☐ **1** ☐ **2** ☐ **3** ☐ **4** ☐ **5** ☐ **6** ☐ **7** ☐ **8** ☐ **9** ☐ **10** ☐
14. Other Activities of Daily Living	On a 0-10 scale, how severe are any problems you have in doing your usual activities, such as your household work, leisure activities, work, study or shopping ?<br>0 means no problems, 10 means completely unable to do or fully dependent on others to help.<br>Now: **0** ☐ **1** ☐ **2** ☐ **3** ☐ **4** ☐ **5** ☐ **6** ☐ **7** ☐ **8** ☐ **9** ☐ **10** ☐<br>Pre-Covid: **0** ☐ **1** ☐ **2** ☐ **3** ☐ **4** ☐ **5** ☐ **6** ☐ **7** ☐ **8** ☐ **9** ☐ **10** ☐
15. Social role	On a 0-10 scale, how severe are any problems you have in caring for family members and/or your interaction with friends that are related to your illness (and not due to the social distancing/lockdown measures) ?<br>0 means no problems, 10 means completely unable to do<br>Now: **0** ☐ **1** ☐ **2** ☐ **3** ☐ **4** ☐ **5** ☐ **6** ☐ **7** ☐ **8** ☐ **9** ☐ **10** ☐

	Pre-Covid: **0** ☐ **1** ☐ **2** ☐ **3** ☐ **4** ☐ **5** ☐ **6** ☐ **7** ☐ **8** ☐ **9** ☐ **10** ☐

What is your employment situation and has your illness affected your ability to do your usual work?<br>Occupation: ________________________<br>Employment status before Covid-19 Lockdown: ________________________<br>Employment status before you became ill: ________________________<br>Employment status now: ________________________
Do you think your family or carer would have anything to add from their perspective?
Are you experiencing any other new problems since your illness we haven't mentioned? Rate the severity of the problem (0 being not present, 10 being severe and life disturbing)<br>Palpitations: **0** ☐ **1** ☐ **2** ☐ **3** ☐ **4** ☐ **5** ☐ **6** ☐ **7** ☐ **8** ☐ **9** ☐ **10** ☐<br>Dizziness/ falls: **0** ☐ **1** ☐ **2** ☐ **3** ☐ **4** ☐ **5** ☐ **6** ☐ **7** ☐ **8** ☐ **9** ☐ **10** ☐<br>Weakness: **0** ☐ **1** ☐ **2** ☐ **3** ☐ **4** ☐ **5** ☐ **6** ☐ **7** ☐ **8** ☐ **9** ☐ **10** ☐<br>Sleep problems: **0** ☐ **1** ☐ **2** ☐ **3** ☐ **4** ☐ **5** ☐ **6** ☐ **7** ☐ **8** ☐ **9** ☐ **10** ☐<br>Fever: **0** ☐ **1** ☐ **2** ☐ **3** ☐ **4** ☐ **5** ☐ **6** ☐ **7** ☐ **8** ☐ **9** ☐ **10** ☐<br>Skin rash: **0** ☐ **1** ☐ **2** ☐ **3** ☐ **4** ☐ **5** ☐ **6** ☐ **7** ☐ **8** ☐ **9** ☐ **10** ☐<br><br>Other symptoms – free text
How good or bad is your health overall?<br><br>NB: PLEASE NOTE THAT THIS QUESTION IS SCORED IN THE OPPOSITE DIRECTION TO THE REST OF THE QUESTIONS IN THIS QUESTIONNAIRE.<br><br>For this question, a score of 10 means the BEST health you can imagine. 0 means the WORST health you can imagine.<br><br>a) Now: WORST HEALTH 0 ☐ 1 ☐ 2 ☐ 3 ☐ 4 ☐ 5 ☐ 6 ☐ 7 ☐ 8 ☐ 9 ☐ 10 ☐ BEST HEALTH<br>b) Pre-Covid: WORST HEALTH 0 ☐ 1 ☐ 2 ☐ 3 ☐ 4 ☐ 5 ☐ 6 ☐ 7 ☐ 8 ☐ 9 ☐ 10 ☐ BEST HEALTH
