# Supplementary material for: Translation and cultural adaptation of the COVID-19 Yorkshire Rehabilitation Scale into German
Source: Front Med (Lausanne). 2024 Aug 30;11:1401491. doi: 10.3389/fmed.2024.1401491 (PMC11409085; doi:10.3389/fmed.2024.1401491)
Supplement: Supplementary file 2 [file Data_Sheet_2.pdf]

COVID-19 Yorkshire Rehabilitation Scale (C19-YRS)

Selbstbeurteilungsbogen zur Erfassung von anhaltenden COVID-19 Symptomen

©Manoj Sivan

Ins Deutsche übersetzt von Lisa Sperl, Erika Mosor, Valentin Ritschl und Tanja Stamm

Patient*innen ID:

Ausfülldatum (tt.mm.jjjj): Uhrzeit (hh:mm):

Zweck dieses Fragebogens ist es, herauszufinden, ob Sie aktuell Probleme haben, die mit Ihrer kürzlich stattgefundenen COVID-19 Erkrankung zusammenhängen. Wir werden diese Informationen nutzen, um Ihre Symptome zu beobachten, entsprechende Behandlungen anzubieten und das Ansprechen auf die Behandlungen zu beurteilen.

Die Beantwortung dieses Fragebogens dauert etwa 15 Minuten. Wenn es Themen gibt, über die Sie keine Auskunft geben möchten, dann lassen Sie die entsprechende Frage weg.

Einstiegsfragen:

Hatten Sie im Zusammenhang mit COVID-19 medizinische Probleme, die einen Krankenhausaufenthalt erforderlich machten? **Ja** □ **Nein** □<br><br>Könnten Sie diese bitte konkretisieren:
Haben Sie andere Gesundheitsdienstleistungen zur Behandlung von COVID-19 Symptomen in Anspruch genommen (z.B. Hausärzt*in, Lungenfachärzt*in, 1450 Corona Hotline)?<br>**Ja** □ **Nein** □<br><br>Könnten Sie diese bitte konkretisieren:

<table>
<tr><td colspan="3">Bitte beantworten Sie die folgenden Fragen so genau wie möglich.
“Jetzt” bezieht sich darauf, wie Sie sich jetzt/ diese Woche fühlen.
“Vor-COVID” bezieht sich darauf, wie Sie sich fühlten, bevor Sie an COVID-19 erkrankt sind.</td></tr>
<tr><td rowspan="5">1. Atemlosigkeit/ Kurzatmigkeit</td><td colspan="3">Auf einer Skala von 0 bis 10, wie schwer würden Sie eine (eventuell vorhandene) Atemlosigkeit/ Kurzatmigkeit einschätzen?

Bewerten Sie den Schweregrad dieses Problems (zwischen 0 - nicht vorhanden und 10 - schwerwiegend und Ihr Leben beeinträchtigend).

(keine Antwort (k/a), wenn Sie die unten angeführten Tätigkeiten nicht ausüben)</td></tr>
<tr><td></td><td>Vor-COVID</td><td>Jetzt</td></tr>
<tr><td>a) In Ruhe</td><td>0-10: ____</td><td>0-10: ____</td></tr>
<tr><td>b) Beim Anziehen</td><td>0-10: ____
k/a ☐</td><td>0-10: ____
k/a ☐</td></tr>
<tr><td>c) Beim Treppen hinaufsteigen</td><td>0-10: ____
k/a ☐</td><td>0-10: ____
k/a ☐</td></tr>
<tr><td>2. Husten/ Empfindlichkeit des Rachens/ Veränderung der Stimme</td><td colspan="3">Haben Sie eines der folgenden Symptome, das seit der Erkrankung neu aufgetreten ist?

• Husten, Empfindlichkeit im Hals Ja ☐ Nein ☐
• Veränderung der Stimme Ja ☐ Nein ☐
• Geräuschvolles Atmen Ja ☐ Nein ☐
Welches dieser drei Symptome belastet Sie am meisten?
[ ]

Bewerten Sie den Schweregrad dieses Problems (zwischen 0 - nicht vorhanden und 10 - schwerwiegend und Ihr Leben beeinträchtigend).

Jetzt: 0 ☐ 1 ☐ 2 ☐ 3 ☐ 4 ☐ 5 ☐ 6 ☐ 7 ☐ 8 ☐ 9 ☐ 10 ☐
Vor-Covid: 0 ☐ 1 ☐ 2 ☐ 3 ☐ 4 ☐ 5 ☐ 6 ☐ 7 ☐ 8 ☐ 9 ☐ 10 ☐</td></tr>
<tr><td>3. Schlucken/ Ernährung</td><td colspan="3">Haben Sie Schwierigkeiten beim Essen, Trinken oder Schlucken, z. B. Husten, Würgen, oder vermeiden Sie bestimmte Speisen oder Getränke?

Ja ☐ Nein ☐

Bewerten Sie den Schweregrad des Schluckproblems (zwischen 0 - nicht vorhanden und 10 - schwerwiegend und Ihr Leben beeinträchtigend).

Jetzt: 0 ☐ 1 ☐ 2 ☐ 3 ☐ 4 ☐ 5 ☐ 6 ☐ 7 ☐ 8 ☐ 9 ☐ 10 ☐
Vor-Covid: 0 ☐ 1 ☐ 2 ☐ 3 ☐ 4 ☐ 5 ☐ 6 ☐ 7 ☐ 8 ☐ 9 ☐ 10 ☐</td></tr>
</table>

	Sind Sie oder Ihre Familie besorgt, dass Sie aufgrund von Covid-19 weiterhin Gewicht verlieren oder Ernährungsprobleme haben? **Ja** □ **Nein** □
**4. Chronische Erschöpfung/ Müdigkeit**	Fühlen Sie sich rascher müde/erschöpft als vor Ihrer Krankheit? **Ja** □ **Nein** □<br><br>Bewerten Sie den Schweregrad der Erschöpfung/ Müdigkeit (zwischen 0 - nicht vorhanden und 10 - schwerwiegend und Ihr Leben beeinträchtigend).<br><br>Jetzt: **0** □ **1** □ **2** □ **3** □ **4** □ **5** □**6** □ **7** □ **8** □ **9** □ **10** □<br>Vor-Covid: **0** □ **1** □ **2** □ **3** □ **4** □ **5** □**6** □ **7** □ **8** □ **9** □ **10** □
**5. Kontinenz**	Haben Sie seit Ihrer Erkrankung <u>neu</u> auftretende Probleme mit:<br><br>• der Kontrolle Ihres Darms **Ja** □ **Nein** □<br>• der Kontrolle Ihrer Blase **Ja** □ **Nein** □<br><br>Welches dieser beiden Symptome belastet Sie mehr?<br><br>Bewerten Sie den Schweregrad dieses Problems (zwischen 0 - nicht vorhanden und 10 - schwerwiegend und Ihr Leben beeinträchtigend).<br><br>Jetzt: **0** □ **1** □ **2** □ **3** □ **4** □ **5** □**6** □ **7** □ **8** □ **9** □ **10** □<br>Vor-Covid: **0** □ **1** □ **2** □ **3** □ **4** □ **5** □**6** □ **7** □ **8** □ **9** □ **10** □
**6. Schmerzen/ Unbehagen**	Haben Sie seit der Erkrankung neu auftretende Schmerzen? **Ja** □ **Nein** □<br><br>Wenn Ja:<br><br>• Brustschmerzen **Ja** □ **Nein** □<br>• Gelenksschmerzen **Ja** □ **Nein** □<br>• Muskelschmerzen **Ja** □ **Nein** □<br>• Kopfschmerzen **Ja** □ **Nein** □<br>• Bauchschmerzen **Ja** □ **Nein** □<br>• Andere Schmerzen **Ja** □ **Nein** □<br>Welche dieser Schmerzen haben Sie in der letzten Woche am meisten belastet?<br><br>Bewerten Sie den Schweregrad dieses Problems (zwischen 0 - keine Schmerzen oder Beschwerden und 10 - schwerwiegende und Ihr Leben beeinträchtigende Schmerzen).<br><br>Jetzt: **0** □ **1** □ **2** □ **3** □ **4** □ **5** □**6** □ **7** □ **8** □ **9** □ **10** □<br>Vor-Covid: **0** □ **1** □ **2** □ **3** □ **4** □ **5** □**6** □ **7** □ **8** □ **9** □ **10** □

**7. Kognition**	Hatten Sie seit Ihrer Erkrankung neue oder verstärkt Schwierigkeiten mit:<br><br>• Konzentration **Ja □ Nein □**<br>• Kurzzeitgedächtnis **Ja □ Nein □**<br>• Organisation im Alltag **Ja □ Nein □**<br>Welches dieser drei Symptome belastet Sie am meisten?<br><br>Bewerten Sie den Schweregrad dieses Problems (zwischen 0 - nicht vorhanden und 10 - schwerwiegend und Ihr Leben beeinträchtigend).<br><br>Jetzt: **0 □ 1 □ 2 □ 3 □ 4 □ 5 □6 □ 7 □ 8 □ 9 □ 10 □**<br>Vor-Covid: **0 □ 1 □ 2 □ 3 □ 4 □ 5 □6 □ 7 □ 8 □ 9 □ 10 □**
**8. Angstzustände**	Auf einer Skala von 0 bis 10, wie stark würden Sie Ihre (möglichen) derzeitigen Angstzustände einschätzen?<br><br>0 bedeutet, ich habe keine Angst, 10 bedeutet, ich habe extreme Angstzustände.<br><br>Jetzt: **0 □ 1 □ 2 □ 3 □ 4 □ 5 □6 □ 7 □ 8 □ 9 □ 10 □**<br>Vor-Covid: **0 □ 1 □ 2 □ 3 □ 4 □ 5 □6 □ 7 □ 8 □ 9 □ 10 □**
**9. Depression**	Auf einer Skala von 0 bis 10, wie schwer würden Sie eine (eventuell vorhandene) Depression einschätzen?<br><br>0 bedeutet, dass ich nicht depressiv bin, 10 bedeutet, dass ich eine sehr starke Depression habe.<br><br>Jetzt: **0 □ 1 □ 2 □ 3 □ 4 □ 5 □6 □ 7 □ 8 □ 9 □ 10 □**<br>Vor-Covid: **0 □ 1 □ 2 □ 3 □ 4 □ 5 □6 □ 7 □ 8 □ 9 □ 10 □**<br><br>Denken Sie gegenwärtig daran, sich in irgendeiner Weise selbst zu verletzen?<br><br>**Ja □ Nein □**
**10. Screening für eine Posttraumatische Belastungsstörung**	a) Hatten Sie irgendwelche unangenehmen Erinnerungen an Ihre Krankheit **oder** Ihren Krankenhausaufenthalt, während Sie wach waren, also nicht im Schlaf?<br>**Ja □ Nein □**<br>b) Hatten Sie unangenehme Träume über Ihre Krankheit **oder** Ihren Krankenhausaufenthalt? **Ja □ Nein□**<br>c) Haben Sie versucht, Gedanken oder Gefühle über Ihre Krankheit **oder** die Aufnahme ins Krankenhaus zu vermeiden? **Ja □ Nein □**

	Bewerten Sie den Schweregrad dieser Belastungsprobleme (zwischen 0 - nicht vorhanden und 10 - schwerwiegend und Ihr Leben beeinträchtigend)<br><br>Jetzt: **0** □ **1** □ **2** □ **3** □ **4** □ **5** □**6** □ **7** □ **8** □ **9** □ **10** □<br><br>Vor-Covid: **0** □ **1** □ **2** □ **3** □ **4** □ **5** □**6** □ **7** □ **8** □ **9** □ **10** □
**11. Kommunikation**	Haben Sie seit Ihrer Erkrankung neue oder verstärkt Schwierigkeiten in der Kommunikation/bei der Wortfindung/ beim Verstehen anderer? **Ja** □ **Nein** □<br><br>Bewerten Sie den Schweregrad des Kommunikationsproblems (zwischen 0 - nicht vorhanden und 10 - schwerwiegend und Ihr Leben beeinträchtigend)<br><br>Jetzt: **0** □ **1** □ **2** □ **3** □ **4** □ **5** □**6** □ **7** □ **8** □ **9** □ **10** □<br>Vor-Covid: **0** □ **1** □ **2** □ **3** □ **4** □ **5** □**6** □ **7** □ **8** □ **9** □ **10** □
**12. Mobilität**	Auf einer Skala von 0 bis 10, wie stark sind die Probleme, die Sie beim Gehen haben?<br>*Oder bei der Fortbewegung, wenn Sie einen Rollstuhl oder ein anderes Hilfsmittel benützen*<br>Zwischen 0 - nicht vorhanden und 10 - schwerwiegende Probleme in der Fortbewegung.<br><br>Jetzt: **0** □ **1** □ **2** □ **3** □ **4** □ **5** □**6** □ **7** □ **8** □ **9** □ **10** □<br>Vor-Covid: **0** □ **1** □ **2** □ **3** □ **4** □ **5** □**6** □ **7** □ **8** □ **9** □ **10** □
**13. Körperpflege**	Auf einer Skala von 0 bis 10, wie schwer sind Ihre Probleme bei der Körperpflege, z. B. beim Benutzen der Toilette, beim Waschen und Anziehen Ihrer Kleidung?<br><br>0 bedeutet, dass es keine Probleme gibt, 10 bedeutet, dass man schwerwiegende Probleme hat oder vollständig auf die Hilfe anderer angewiesen ist.<br><br>Jetzt: **0** □ **1** □ **2** □ **3** □ **4** □ **5** □**6** □ **7** □ **8** □ **9** □ **10** □<br>Vor-Covid: **0** □ **1** □ **2** □ **3** □ **4** □ **5** □**6** □ **7** □ **8** □ **9** □ **10** □
**14. Andere Aktivitäten des täglichen Lebens**	Auf einer Skala von 0 bis 10, wie schwerwiegend sind die Probleme, die Sie bei Ihren üblichen Aktivitäten haben, wie z. B. bei der Hausarbeit, bei Freizeitaktivitäten, bei der Arbeit, beim Lernen oder beim Einkaufen?<br><br>0 bedeutet, dass es keine Probleme gibt, 10 bedeutet, dass man schwerwiegende Probleme hat oder vollständig auf die Hilfe anderer angewiesen ist.<br><br>Jetzt: **0** □ **1** □ **2** □ **3** □ **4** □ **5** □**6** □ **7** □ **8** □ **9** □ **10** □

	Vor-Covid: **0** ☐ **1** ☐ **2** ☐ **3** ☐ **4** ☐ **5** ☐**6** ☐ **7** ☐ **8** ☐ **9** ☐ **10** ☐
**15. Soziale Rolle**	Wie schwerwiegend sind auf einer Skala von 0 bis 10 die Probleme, die Sie zum Beispiel im Kontakt mit Familienmitgliedern oder mit Freund*innen haben? Gibt es hier Einschränkungen ih ihrem sozialen Leben, die mit Ihren anhaltenden Symptomen zusammenhängen (und nicht auf die COVID-19 Maßnahmen zur sozialen Distanzierung/ Lockdown zurückzuführen sind)?<br><br>0 bedeutet keine Probleme, 10 bedeutet schwerwiegende Probleme<br><br>Jetzt: **0** ☐ **1** ☐ **2** ☐ **3** ☐ **4** ☐ **5** ☐**6** ☐ **7** ☐ **8** ☐ **9** ☐ **10** ☐<br>Vor-Covid: **0** ☐ **1** ☐ **2** ☐ **3** ☐ **4** ☐ **5** ☐**6** ☐ **7** ☐ **8** ☐ **9** ☐ **10** ☐

Wie ist Ihr aktueller Beschäftigungsstatus? Hat Ihre Krankheit Ihre Fähigkeit beeinträchtigt, Ihrer üblichen Arbeit nachzugehen?<br><br>Beruf: _______________________<br><br>Beschäftigungsstatus vor der Covid-19 Pandemie: _______________________<br><br>Beschäftigungsstatus vor Ihrer Covid-19 Erkrankung: _______________________<br><br>Aktueller Beschäftigungsstatus: _______________________
Glauben Sie, dass Ihre Familie/Betreuungspersonen aus ihrer Sicht etwas zu diesem Fragebogen hinzufügen möchten?
Haben Sie seit Ihrer Krankheit andere neue Probleme, die wir nicht erwähnt haben? Bewerten Sie den Schweregrad des jeweiligen Problems (zwischen 0 - nicht vorhanden und 10 - schwerwiegend und Ihr Leben beeinträchtigend).<br><br>Herzrasen/ Herzflattern: **0** ☐ **1** ☐ **2** ☐ **3** ☐ **4** ☐ **5** ☐**6** ☐ **7** ☐ **8** ☐ **9** ☐ **10** ☐<br>Schwindel/ Stürze: **0** ☐ **1** ☐ **2** ☐ **3** ☐ **4** ☐ **5** ☐**6** ☐ **7** ☐ **8** ☐ **9** ☐ **10** ☐<br>Schwäche: **0** ☐ **1** ☐ **2** ☐ **3** ☐ **4** ☐ **5** ☐**6** ☐ **7** ☐ **8** ☐ **9** ☐ **10** ☐<br>Schlafstörungen: **0** ☐ **1** ☐ **2** ☐ **3** ☐ **4** ☐ **5** ☐**6** ☐ **7** ☐ **8** ☐ **9** ☐ **10** ☐<br>Fieber: **0** ☐ **1** ☐ **2** ☐ **3** ☐ **4** ☐ **5** ☐**6** ☐ **7** ☐ **8** ☐ **9** ☐ **10** ☐<br>Hautausschlag: **0** ☐ **1** ☐ **2** ☐ **3** ☐ **4** ☐ **5** ☐**6** ☐ **7** ☐ **8** ☐ **9** ☐ **10** ☐

Andere Symptome:
Wie gut oder schlecht schätzen Sie Ihren Gesundheitszustand insgesamt ein?<br><br>HINWEIS: BITTE BEACHTEN SIE, DASS DIESE FRAGE IN UMGEKEHRTER RICHTUNG WIE DIE ÜBRIGEN FRAGEN IN DIESEM FRAGEBOGEN BEWERTET WIRD.<br><br>Bei dieser Frage bedeutet ein Wert von 10 den BESTEN Gesundheitszustand, den Sie sich vorstellen können. 0 bedeutet en schlechtesten Gesundheitszustand, den Sie sich vorstellen können.<br><br>a) Jetzt: 0 ☐ 1 ☐ 2 ☐ 3 ☐ 4 ☐ 5 ☐ 6 ☐ 7 ☐ 8 ☐ 9 ☐ 10 ☐<br>b) Vor-Covid: 0 ☐ 1 ☐ 2 ☐ 3 ☐ 4 ☐ 5 ☐ 6 ☐ 7 ☐ 8 ☐ 9 ☐ 10 ☐
