# Supplementary material for: Translation and cultural adaptation of the COVID-19 Yorkshire Rehabilitation Scale into German
Source: Front Med (Lausanne). 2024 Aug 30;11:1401491. doi: 10.3389/fmed.2024.1401491 (PMC11409085; doi:10.3389/fmed.2024.1401491)
Supplement: Supplementary file 3 [file Data_Sheet_3.pdf]

Cognitive Interview Protocol for the Pre-testing of the COVID-19 Yorkshire Rehabilitation Scale (C19-YRS) including administered probes

**Subsection 1 – Einleitung**
**<u>Text 1:</u>** COVID-19 Yorkshire Rehabilitation Screening (C19-YRS)<br><br>Selbstbeurteilungsbogen zur Erfassung von anhaltenden COVID-19 Symptomen
**<u>Q 1:</u>** Patient*innen Code:
**<u>Q 2:</u>** Ausfülldatum (tt.mm.jjjj):
**<u>Q 3:</u>** Uhrzeit (hh:mm):
**<u>Text 2:</u>** Wir nehmen Kontakt zu Personen auf, die nach einer COVID-19 Erkrankung anhaltende gesundheitliche Probleme haben. Zweck dieses Fragebogens ist es, herauszufinden, ob Sie aktuell Probleme haben, die mit Ihrer kürzlich stattgefundenen COVID-19 Erkrankung zusammenhängen. Wir werden diese Informationen nutzen, um Ihre Symptome zu beobachten, entsprechende Behandlungen anzubieten und das Ansprechen auf die Behandlungen zu beurteilen.<br><br>Die Beantwortung dieses Fragebogens dauert etwa 15 Minuten. Wenn es Themen gibt, über die Sie nicht sprechen möchten, dann antworten Sie bitte nicht.
**Probes for Subsection 1:**<br><br><u>1.1 General:</u> Wie haben Sie die Einleitung gefunden?<br><br><u>1.2 Comprehension:</u> Was haben Sie unter dem Begriff "anhaltende gesundheitliche Probleme" verstanden?
**Subsection 2 – Einstiegsfragen**
**<u>Q 4:</u>** Hatten Sie im Zusammenhang mit COVID-19 medizinischen Probleme, die einen Krankenhausaufenthalt erforderlich machten? Ja ☐ Nein ☐<br><br>Details:
**<u>Q 5:</u>** Haben Sie andere Gesundheitsdienstleistungen zur Behandlung von COVID-19 Symptomen in Anspruch genommen (z.B. Allgemeinmediziner*in/ Hausärzt*in)? Ja ☐ Nein ☐<br><br>Details:
**Probes for Subsection 2:**<br><br><u>2.1 General:</u> Wie haben Sie diese Fragen gefunden?<br><br><u>2.2 Comprehension:</u> Was haben Sie unter dem Begriff "Gesundheitsdienstleistungen" verstanden?<br><br><u>2.3 Retrieval:</u> War es leicht oder schwer für Sie, sich an diese Informationen zu erinnern?

2.4 Response: Waren die Antwortmöglichkeiten für diese Fragen passend oder eher unpassend?

Subsection 3 – Symptome

Text 3: *Bitte beantworten Sie die folgenden Fragen so genau wie möglich.*

"Jetzt" bezieht sich darauf, wie Sie sich jetzt/ diese Woche fühlen.

"Vor-COVID" bezieht sich darauf, wie Sie sich fühlten, bevor Sie an COVID-19 erkrankt sind.

Q 6: 1. Atemlosigkeit/ Kurzatmigkeit

Auf einer Skala von 0 bis 10, wie schwer würden Sie eine (eventuell vorhandene) Atemlosigkeit/ Kurzatmigkeit einschätzen?

Bewerten Sie den Schweregrad dieses Problems (zwischen 0 - nicht vorhanden und 10 - schwerwiegend und Ihr Leben beeinträchtigend).

(keine Antwort (k/a), wenn Sie die unten angeführten Tätigkeiten nicht ausüben)

		Vor-Covid	Jetzt
a)	In Ruhe	0-10: ____	0-10: ____
b)	Beim Anziehen	0-10: ____ k/a ☐	0-10: ____k/a ☐
c)	Beim Treppen hinaufsteigen	0-10: ____k/a ☐	0-10: ____k/a ☐

Q 7: 2. Husten/ Empfindlichkeit des Rachens/ Veränderung der Stimme

Haben Sie eines der folgenden Symptome, das seit der Erkrankung neu aufgetreten ist?

- Husten, Empfindlichkeit im Hals Ja ☐ Nein ☐
- Veränderung der Stimme Ja ☐ Nein ☐
- Geräuschvolles Atmen Ja ☐ Nein ☐

Welches dieser drei Symptome belastet Sie am meisten?

Bewerten Sie den Schweregrad dieses Problems (zwischen 0 - nicht vorhanden und 10 - schwerwiegend und Ihr Leben beeinträchtigend).

Jetzt: 0 ☐ 1 ☐ 2 ☐ 3 ☐ 4 ☐ 5 ☐ 6 ☐ 7 ☐ 8 ☐ 9 ☐ 10 ☐

Vor-Covid: 0 ☐ 1 ☐ 2 ☐ 3 ☐ 4 ☐ 5 ☐ 6 ☐ 7 ☐ 8 ☐ 9 ☐ 10 ☐

Q 8: 3. Schlucken/ Ernährung

Haben Sie Schwierigkeiten beim Essen, Trinken oder Schlucken, z. B. Husten, Würgen, der vermeiden Sie bestimmte Speisen oder Getränke? Ja ☐ Nein ☐

Bewerten Sie den Schweregrad des Schluckproblems (zwischen 0 - nicht vorhanden und 10 - schwerwiegend und Ihr Leben beeinträchtigend).

Jetzt: 0 ☐ 1 ☐ 2 ☐ 3 ☐ 4 ☐ 5 ☐ 6 ☐ 7 ☐ 8 ☐ 9 ☐ 10 ☐

Vor-Covid: 0 ☐ 1 ☐ 2 ☐ 3 ☐ 4 ☐ 5 ☐ 6 ☐ 7 ☐ 8 ☐ 9 ☐ 10 ☐

Sind Sie oder Ihre Familie besorgt, dass Sie aufgrund von Covid-19 weiterhin Gewicht

verlieren oder Ernährungsprobleme haben? Ja ☐ Nein ☐

Q 9: 4. Chronische Erschöpfung/ Müdigkeit

Fühlen Sie sich rascher müde/erschöpft als vor Ihrer Krankheit? Ja ☐ Nein ☐

Bewerten Sie den Schweregrad der Erschöpfung/ Müdigkeit (zwischen 0 - nicht vorhanden und 10 - schwerwiegend und Ihr Leben beeinträchtigend).

Jetzt: 0 ☐ 1 ☐ 2 ☐ 3 ☐ 4 ☐ 5 ☐ 6 ☐ 7 ☐ 8 ☐ 9 ☐ 10 ☐

Vor-Covid: 0 ☐ 1 ☐ 2 ☐ 3 ☐ 4 ☐ 5 ☐ 6 ☐ 7 ☐ 8 ☐ 9 ☐ 10 ☐

Q 10: 5. Kontinenz

Haben Sie seit Ihrer Erkrankung neu auftretende Probleme mit:

- der Kontrolle Ihres Darms Ja ☐ Nein ☐
- der Kontrolle Ihrer Blase Ja ☐ Nein ☐

Welches dieser beiden Symptome belastet Sie mehr?

Bewerten Sie den Schweregrad dieses Problems (zwischen 0 - nicht vorhanden und 10 - schwerwiegend und Ihr Leben beeinträchtigend).

Jetzt: 0 ☐ 1 ☐ 2 ☐ 3 ☐ 4 ☐ 5 ☐ 6 ☐ 7 ☐ 8 ☐ 9 ☐ 10 ☐

Vor-Covid: 0 ☐ 1 ☐ 2 ☐ 3 ☐ 4 ☐ 5 ☐ 6 ☐ 7 ☐ 8 ☐ 9 ☐ 10 ☐

Q 11: 6. Schmerzen/ Unbehagen

Haben Sie seit der Erkrankung neu auftretende Schmerzen? Ja ☐ Nein ☐

Wenn Ja:

- Brustschmerzen Ja ☐ Nein ☐
- Gelenksschmerzen Ja ☐ Nein ☐
- Muskelschmerzen Ja ☐ Nein ☐
- Kopfschmerzen Ja ☐ Nein ☐
- Bauchschmerzen Ja ☐ Nein ☐
- Andere Schmerzen Ja ☐ Nein ☐

Welche dieser Schmerzen haben Sie in der letzten Woche am meisten belastet?

Bewerten Sie den Schweregrad dieses Problems (zwischen 0 - keine Schmerzen oder Beschwerden und 10 - schwerwiegende und Ihr Leben beeinträchtigende Schmerzen).

Jetzt: 0 ☐ 1 ☐ 2 ☐ 3 ☐ 4 ☐ 5 ☐ 6 ☐ 7 ☐ 8 ☐ 9 ☐ 10 ☐

Vor-Covid: 0 ☐ 1 ☐ 2 ☐ 3 ☐ 4 ☐ 5 ☐ 6 ☐ 7 ☐ 8 ☐ 9 ☐ 10 ☐

Q 12: 7. Kognition

Hatten Sie seit Ihrer Erkrankung neue oder verstärkt Schwierigkeiten mit:

- Konzentration Ja ☐ Nein ☐
- Kurzzeitgedächtnis Ja ☐ Nein ☐
- Organisation im Alltag Ja ☐ Nein ☐

Welches dieser drei Symptome belastet Sie am meisten?

Bewerten Sie den Schweregrad dieses Problems (zwischen 0 - nicht vorhanden und 10 - schwerwiegend und Ihr Leben beeinträchtigend).

Jetzt: 0 ☐ 1 ☐ 2 ☐ 3 ☐ 4 ☐ 5 ☐ 6 ☐ 7 ☐ 8 ☐ 9 ☐ 10 ☐

Vor-Covid: 0 ☐ 1 ☐ 2 ☐ 3 ☐ 4 ☐ 5 ☐ 6 ☐ 7 ☐ 8 ☐ 9 ☐ 10 ☐

Q 13: 8. Angstzustände

Auf einer Skala von 0 bis 10, wie stark würden Sie Ihre (möglichen) derzeitigen Angstzustände einschätzen?

0 bedeutet, ich habe keine Angst, 10 bedeutet, ich habe extreme Angstzustände.

Jetzt: 0 ☐ 1 ☐ 2 ☐ 3 ☐ 4 ☐ 5 ☐ 6 ☐ 7 ☐ 8 ☐ 9 ☐ 10 ☐<br><br>Vor-Covid: 0 ☐ 1 ☐ 2 ☐ 3 ☐ 4 ☐ 5 ☐ 6 ☐ 7 ☐ 8 ☐ 9 ☐ 10 ☐
**Q 14:** 9. Depression<br><br>Auf einer Skala von 0 bis 10, wie schwer würden Sie eine (eventuell vorhandene) Depression einschätzen?<br><br>0 bedeutet, dass ich nicht depressiv bin, 10 bedeutet, dass ich eine sehr starke Depression habe.<br><br>Jetzt: 0 ☐ 1 ☐ 2 ☐ 3 ☐ 4 ☐ 5 ☐ 6 ☐ 7 ☐ 8 ☐ 9 ☐ 10 ☐<br><br>Vor-Covid: 0 ☐ 1 ☐ 2 ☐ 3 ☐ 4 ☐ 5 ☐ 6 ☐ 7 ☐ 8 ☐ 9 ☐ 10 ☐<br><br>Denken Sie gegenwärtig daran, sich in irgendeiner Weise selbst zu verletzen?<br><br>Ja ☐ Nein ☐
**Q 15:** 10. Screening für eine Posttraumatische Belastungsstörung<br><br>a) Hatten Sie irgendwelche ungewollten Erinnerungen an Ihre Krankheit oder Ihren Krankenhausaufenthalt, während Sie wach waren, also nicht im Schlaf?<br><br>Ja ☐ Nein ☐<br><br>b) Hatten Sie unangenehme Träume über Ihre Krankheit oder Ihren Krankenhausaufenthalt? Ja ☐ Nein☐<br><br>c) Haben Sie versucht, Gedanken oder Gefühle über Ihre Krankheit oder die Aufnahme ins Krankenhaus zu vermeiden? Ja ☐ Nein ☐<br><br>Bewerten Sie den Schweregrad dieser Belastungsprobleme (zwischen 0 - nicht vorhanden und 10 - schwerwiegend und Ihr Leben beeinträchtigend)<br><br>Jetzt: 0 ☐ 1 ☐ 2 ☐ 3 ☐ 4 ☐ 5 ☐ 6 ☐ 7 ☐ 8 ☐ 9 ☐ 10 ☐<br><br>Vor-Covid: 0 ☐ 1 ☐ 2 ☐ 3 ☐ 4 ☐ 5 ☐ 6 ☐ 7 ☐ 8 ☐ 9 ☐ 10 ☐
**Q 16:** 11. Kommunikation<br><br>Haben Sie seit Ihrer Erkrankung neue oder verstärkt Schwierigkeiten in der Kommunikation/bei der Wortfindung/ beim Verstehen anderer?<br><br>Ja ☐ Nein ☐<br><br>Bewerten Sie den Schweregrad des Kommunikationsproblems (zwischen 0 - nicht vorhanden und 10 - schwerwiegend und Ihr Leben beeinträchtigend)<br><br>Jetzt: 0 ☐ 1 ☐ 2 ☐ 3 ☐ 4 ☐ 5 ☐ 6 ☐ 7 ☐ 8 ☐ 9 ☐ 10 ☐<br><br>Vor-Covid: 0 ☐ 1 ☐ 2 ☐ 3 ☐ 4 ☐ 5 ☐ 6 ☐ 7 ☐ 8 ☐ 9 ☐ 10 ☐
**Q 17:** 12. Mobilität<br><br>Auf einer Skala von 0 bis 10, wie stark sind die Probleme, die Sie beim Gehen haben?<br><br>*Oder bei der Fortbewegung, wenn Sie einen Rollstuhl oder ein anderes Hilfsmittel benützen*<br><br>Zwischen 0 - nicht vorhanden und 10 - schwerwiegende Probleme in der Fortbewegung.<br><br>Jetzt: 0 ☐ 1 ☐ 2 ☐ 3 ☐ 4 ☐ 5 ☐ 6 ☐ 7 ☐ 8 ☐ 9 ☐ 10 ☐<br><br>Vor-Covid: 0 ☐ 1 ☐ 2 ☐ 3 ☐ 4 ☐ 5 ☐ 6 ☐ 7 ☐ 8 ☐ 9 ☐ 10 ☐
**Q 18:** 13. Körperpflege

Auf einer Skala von 0 bis 10, wie schwer sind Ihre Probleme bei der Körperpflege, z. B. beim Benutzen der Toilette, beim Waschen und Anziehen Ihrer Kleidung?

0 bedeutet, dass es keine Probleme gibt, 10 bedeutet, dass man schwerwiegende Probleme hat oder vollständig auf die Hilfe anderer angewiesen ist.

Jetzt: 0 ☐ 1 ☐ 2 ☐ 3 ☐ 4 ☐ 5 ☐ 6 ☐ 7 ☐ 8 ☐ 9 ☐ 10 ☐

Vor-Covid: 0 ☐ 1 ☐ 2 ☐ 3 ☐ 4 ☐ 5 ☐ 6 ☐ 7 ☐ 8 ☐ 9 ☐ 10 ☐

Q 19: 14. Andere Aktivitäten des täglichen Lebens

Auf einer Skala von 0 bis 10, wie schwerwiegend sind die Probleme, die Sie bei Ihren üblichen Aktivitäten haben, wie z. B. bei der Hausarbeit, bei Freizeitaktivitäten, bei der Arbeit, beim Lernen oder beim Einkaufen?

0 bedeutet, dass es keine Probleme gibt, 10 bedeutet, dass man schwerwiegende Probleme hat oder vollständig auf die Hilfe anderer angewiesen ist.

Jetzt: 0 ☐ 1 ☐ 2 ☐ 3 ☐ 4 ☐ 5 ☐ 6 ☐ 7 ☐ 8 ☐ 9 ☐ 10 ☐

Vor-Covid: 0 ☐ 1 ☐ 2 ☐ 3 ☐ 4 ☐ 5 ☐ 6 ☐ 7 ☐ 8 ☐ 9 ☐ 10 ☐

Q 20: 15. Soziale Rolle

Wie schwerwiegend sind auf einer Skala von 0 bis 10 die Probleme, die Sie bei der Betreuung von Familienmitgliedern und/oder im Kontakt mit Freund*innen haben, die mit Ihrer Krankheit zusammenhängen (und nicht auf die COVID-19 Maßnahmen zur sozialen Distanzierung/ Lockdown zurückzuführen sind)?

0 bedeutet keine Probleme, 10 bedeutet schwerwiegende Probleme

Jetzt: 0 ☐ 1 ☐ 2 ☐ 3 ☐ 4 ☐ 5 ☐ 6 ☐ 7 ☐ 8 ☐ 9 ☐ 10 ☐

Vor-Covid: 0 ☐ 1 ☐ 2 ☐ 3 ☐ 4 ☐ 5 ☐ 6 ☐ 7 ☐ 8 ☐ 9 ☐ 10 ☐

Probes for Subsection 3:

3.1 General: Wie haben Sie diese Fragen gefunden?

3.2 Comprehension: Waren die Symptome, sowie die Fragestellungen zu den einzelnen Symptomen, leicht oder schwer zu verstehen?

3.3 Retrieval: War es leicht oder schwer sich an den Zustand vor der COVID Erkrankung zu erinnern?

3.4 Response: Waren die Antwortmöglichkeiten für diese Fragen passend oder eher unpassend?

Subsection 4 – Beschäftigungssituation

Q 21: In welchem Beschäftigungsverhältnis stehen Sie? Hat Ihre Krankheit Ihre Fähigkeit beeinträchtigt, Ihrer üblichen Arbeit nachzugehen?

Beruf: ______________________

Beschäftigungsstatus vor der Covid-19 Pandemie: ______________________

Beschäftigungsstatus vor Ihrer Covid-19 Erkrankung: ______________________

Aktueller Beschäftigungsstatus: ______________________

Q 22: Glauben Sie, dass Ihre Familie/Betreuungspersonen aus ihrer Sicht etwas zu diesem Fragebogen hinzufügen möchten?

Probes for Subsection 4:

4.1 General: Wie haben Sie diese Fragen gefunden?

4.2 Comprehension: Was haben Sie unter dem Begriff "Beschäftigungsstatus" verstanden?

4.3 Response: Waren die Antwortmöglichkeiten für diese Fragen passend oder eher unpassend?

Subsection 5 – Andere Symptome

Q 23: Haben Sie seit Ihrer Krankheit andere neue Probleme, die wir nicht erwähnt haben? Bewerten Sie den Schweregrad des jeweiligen Problems (zwischen 0 - nicht vorhanden und 10 - schwerwiegend und Ihr Leben beeinträchtigend).

Herzrasen/ Herzflattern: 0 ☐ 1 ☐ 2 ☐ 3 ☐ 4 ☐ 5 ☐ 6 ☐ 7 ☐ 8 ☐ 9 ☐ 10 ☐

Schwindel/ Stürze: 0 ☐ 1 ☐ 2 ☐ 3 ☐ 4 ☐ 5 ☐ 6 ☐ 7 ☐ 8 ☐ 9 ☐ 10 ☐

Schwäche: 0 ☐ 1 ☐ 2 ☐ 3 ☐ 4 ☐ 5 ☐ 6 ☐ 7 ☐ 8 ☐ 9 ☐ 10 ☐

Schlafstörungen: 0 ☐ 1 ☐ 2 ☐ 3 ☐ 4 ☐ 5 ☐ 6 ☐ 7 ☐ 8 ☐ 9 ☐ 10 ☐

Fieber: 0 ☐ 1 ☐ 2 ☐ 3 ☐ 4 ☐ 5 ☐ 6 ☐ 7 ☐ 8 ☐ 9 ☐ 10 ☐

Hautausschlag: 0 ☐ 1 ☐ 2 ☐ 3 ☐ 4 ☐ 5 ☐ 6 ☐ 7 ☐ 8 ☐ 9 ☐ 10 ☐

Andere Symptome:

Probes for Subsection 5:

5.1 General: Wie haben Sie diese Frage gefunden?

5.2 Comprehension: Waren die Symptome leicht oder schwer zu verstehen?

5.3 Response: Waren die Antwortmöglichkeiten für diese Frage passend oder eher unpassend?

Subsection 6 – Gesundheitszustand

<u>Q 24:</u> Wie gut oder schlecht schätzen Sie Ihren Gesundheitszustand insgesamt ein?

HINWEIS: BITTE BEACHTEN SIE, DASS DIESE FRAGE IN UMGEKEHRTER RICHTUNG WIE DIE ÜBRIGEN FRAGEN IN DIESEM FRAGEBOGEN BEWERTET WIRD.

Bei dieser Frage bedeutet ein Wert von 10 den BESTEN Gesundheitszustand, den Sie sich vorstellen können. 0 bedeutet en schlechtesten Gesundheitszustand, den Sie sich vorstellen können.

a) Jetzt: 0 ☐ 1 ☐ 2 ☐ 3 ☐ 4 ☐ 5 ☐ 6 ☐ 7 ☐ 8 ☐ 9 ☐ 10 ☐

b) Vor-Covid: 0 ☐ 1 ☐ 2 ☐ 3 ☐ 4 ☐ 5 ☐ 6 ☐ 7 ☐ 8 ☐ 9 ☐ 10 ☐

Probes for Subsection 6:

<u>6.1 General:</u> Wie haben Sie diese Abschlussfrage gefunden?

<u>6.2 Comprehension:</u> Was haben Sie unter dem Begriff “Gesundheitszustand” verstanden?

<u>6.3 Retrieval:</u> War es leicht oder schwer sich an den Zustand vor der COVID Erkrankung zu erinnern?

<u>6.4 Response:</u> Waren die Antwortmöglichkeiten für diese Frage passend oder eher unpassend?
